# Factor structure and measurement invariance of the Chinese version of the COVID-19 Phobia Scale in depressive symptoms sample during COVID-19 closure: An exploratory structural equation modeling approach

**DOI:** 10.3389/fpubh.2022.1026294

**Published:** 2022-10-04

**Authors:** Tao Yang, Wei Chen, Qiaodan Lu, Jiaheng Sun

**Affiliations:** ^1^School of Psychology, Guizhou Normal University, Guiyang, China; ^2^Center for Big Data Research in Psychology, Guizhou Normal University, Guiyang, China

**Keywords:** COVID-19 Phobia, exploratory structural equation modeling, measurement invariance, longitudinal invariance, depressive symptoms

## Abstract

The COVID-19 Phobia Scale is an instrument for measuring the phobia of coronavirus. It has a stable four-factor structure and good reliability and validity in other countries and regions. In order to expand related research, this study aims to test the reliability and validity of the COVID-19 Phobia Scale in Chinese adolescents with depressive symptoms. The C19P-SC was translated into Chinese by the method of forward and back translation and tested in 1933 Chinese adolescents with depressive symptoms. Confirmatory factor analysis (CFA) and exploratory structural equation modeling (ESEM) were used to test and compare the four-factor model of the C19P-SC. Then we tested the measurement invariance of the C19P-SC across gender and time. Finally, the reliability was measured with the McDonald's omega coefficients. Consistent with previous studies, the C19P-SC showed a stable four-factor structure. The results showed that ESEM was better than CFA and more reasonable. In addition, the results of multi-group ESEM showed that the C19P-SC met the strict invariance at male and female and partial longitudinal strict invariance. The Mcdonald's omega coefficients of the C19P-SC total scale and each subscale reached the expected acceptable level. In short, the reliability and validity index of C19P-SC has reached an acceptable level, and the measurement invariance of different genders and different time points was established, but the cross-factor phenomenon of individual items was abnormal, and a further revision and testing are still needed.

## Introduction

Corona Virus Disease (COVID-19) is a new acute respiratory infectious disease at the end of 2019, and it has become a major global public health event (1). Compared with severe acute respiratory syndrome coronavirus (SARS) and Middle East respiratory syndrome coronavirus (MERS), COVID-19 is a highly infectious disease (2). On January 20, 2020, Chinese government classified COVID-19 as a Class B infectious disease and treated it as Class A infectious diseases (3). COVID-19 will not only causes physical and mental pain at the individual level of the infected person, but also the psychological stress caused in other populations can also lead to a variety of unfavorable factors (4). On January 26, 2020, the National Health Commission issued guidelines for emergency psychological crisis intervention for people affected by COVID-19 (5). Psychological crisis intervention under the COVID-19 epidemic is not only for confirmed patients, suspected patients, and quarantined people, but also for all medical staff and some social workers (6).

Since the outbreak of the COVID-19, the number of infections has continued to increase, posing a great threat to people's lives and safety (7). The panic and fear of COVID-19 is called “corona phobia,” because the unpredictability of this disease causes people's mental distress (8). Corona phobia can be classified as a special type of DSM-V specific phobia (9). Consistent with studies on SARS and MERS during the previous virus epidemic (10, 11), the COVID-19 pandemic will also cause people to have great fear, anxiety and reaction (9). Evidence of rising levels of people's phobia found in COVID-19-related research (12). Simultaneously, some studies have found that phobia of COVID-19 is an important predictor of increasing people's active isolation, implying that fear plays an important role in preventing COVID-19 (13). As a global public health emergency, the COVID-19 epidemic can lead to psychological crises such as post-traumatic stress disorder, anxiety and depression (14). Adolescent depression is a group of chronic psychological disease syndromes whose symptoms can last until adulthood (15). The Report on National Mental Health Development in China (2019–2020) shows that the detection rate of depression among adolescents is 24.6% (16). Among them, the detection rate of depression in junior high school is about 30%, the detection rate of depression in high school is nearly 40%. The proportion of Chinese adolescents with depression is on the rise, and its incidence is increasing with age (17). Adolescents are in the growing stage of physical and psychological development, and are prone to depression and even suicidal behavior (18). At present, the prevention and control of the COVID-19 in China has become normal, and the work of returning to school for students nationwide is also accelerating (19). Due to the large scope and strong contagion of the COVID-19, students have a fear of COVID-19, and the pressure of isolation can lead to mental disorders such as anxiety and depression (20, 21). Therefore, it is necessary to assess the corona phobia among adolescents with depressive symptoms.

As people become more and more affected by the COVID-19, measurement instruments for corona phobia of COVID-19 have also been developed. Arpaci et al. (9) compiled the COVID-19 Phobia Scale (C19P-S), the C19P-S has 20 items and four dimensions: Psychological, Psycho-Somatic, Economic, Social. So far, the C19P-S has been verified in Turkey, the United States, Korea, and Iran (9, 22–24), all of them showed a stable four-factor structure. However, there is currently no corresponding measurement tool for assessing people's corona phobia in China. For this reason, in order to expand related research, this research aims to test the applicability of the C19P-S in the Chinese cultural context.

Confirmatory factor analysis (CFA) has been widely used in the C19P-S studies (9, 22–24), but CFA was considered to have great limitations when used to test multi-factor measurement models (25). Exploratory structural equation modeling (ESEM) integrates the functions of exploratory factor analysis (EFA) and CFA (26–29). In addition to estimating the load on the main factor of the subject, ESEM also estimates the load of some other factors that are relatively small, which shows us a more realistic situation (30, 31). When validating factor models, ESEM is easier to fit compared with traditional CFA and obtains closer to the real results, thus making some advanced statistical analysis more smoothly (31). Compared with CFA, ESEM seems more reasonable, which has been supported by empirical studies (32). In particular, the advantages of ESEM in testing measurement invariance have also been supported by many empirical studies (26, 27, 31, 33–36).

In order to supplement the research instrument of corona phobia in China and provide more directions for researchers, this study aims to revise the Chinese version of the C19P-S in Chinese adolescents with depressive symptoms and examine its reliability and validity. In addition, few studies have examined an important psychometric feature of C19P-S, namely the measurement invariance of the scale (37). Therefore, this study attempts to use the ESEM method to analyze the structural validity of the C19P-SC. Meanwhile, gender and longitudinal measurement invariance are studied in the ESEM framework. On the one hand, it verifies the structural validity of the C19P-S, on the other hand, it demonstrates the application effect of ESEM method through specific questionnaire data.

## Method

### Participants

This study was complied with the moral standards of the 2013 Helsinki Declaration, and it was approved by the committee of the School of Psychology of Guizhou Normal University. Participants were invited to participate voluntarily between October 2020 and April 2021. In order to facilitate the collection of data for the second test, participants can voluntarily write down their student ID and name when answering the first time. According to the participants' scores on the Patient Health Questionnaire-9 (PHQ-9) (38), including five points and higher in this study. In the first test, after excluding the participant with the PHQ-9 score lower than 5, 1933 adolescents with depressive symptoms were obtained. After 6 months, the questionnaires were send again to participants with student ID and name, 519 participants with PHQ-9 score of five or higher were obtained. Because the screened “depressed adolescents” may also include some adolescents who are not really diagnosed with depression, the screened depressed adolescents in this study refer to non-clinical depressed adolescents, namely adolescents with depressive symptoms.

### Measures

#### The COVID-19 Phobia Scale

The COVID-19 Phobia Scale (C19P-S) was compiled by Arpaci et al. (9) based on the diagnostic criteria of DSM-V's specific phobia (300.29). The scale contains a total of 20 items, used to assess the level of phobia of the COVID-19, using a Likert 5-point scale, “1” means “strongly disagree,” “5” means “strongly agree.” There was no reverse scoring, the higher the total score, the higher the level of phobia symptoms. In previous studies, the subjects were all adults (9, 22–24).

After obtaining authorization from the original author, the English version of the C19P-S was translated. The translation was done collaboratively by two researchers. First, the first researcher will translate the scale from English into Chinese to form the first draft of the Chinese version. Another researcher will translate it back into English and compare it with the original scale. The researchers adjusted the translation of the Chinese version of the first draft based on the comparison of the difference between the back translation scale and the original scale, to ensure that the expression of each item was clearer, and conforms to the Chinese language habits. Finally, the researcher asked a psychologist to review the last Chinese translation of the C19P-S (C19P-SC). The C19P-SC was then pilot-tested with a small sample of thirty school-aged adolescents recruited from elementary, middle, and high schools. Since the school was closed during the COVID-19 epidemic, we contacted the school's psychology teachers by phone or video and sent them the electronic version of the questionnaire, and sent them the electronic file of the questionnaire, and explained the precautions for filling out the questionnaire. With the help of a psychology teacher, the C19P-SC test was completed on thirty adolescents, and most of the adolescents confirmed that all items on the C19P-SC were easy to read and understand, and it took about 10 min to complete.

#### The patient health questionnaire-9

PHQ-9 is a concise self-evaluation questionnaire for depression compiled by Kroenke et al. (38). The nine items of this questionnaire are based on the nine diagnostic criteria in the DSM-IV major depressive episode (32.2) diagnostic criteria. The items are rated on a 4-point Likert scale ranging from 0 (not at all) to three (nearly every day), and no reverse scoring. Its total is the sum of the scores of each item, the theoretical score is 0–27 points, and the scores of 5, 10, and 15 represent mild, moderate, and moderately severe depression, respectively. In this study, the McDonald's omega coefficient was 0.735.

#### Procedure

Since the school is still in a closed period, after obtaining the informed consent of the school, students' parents and teachers, paper questionnaires were sent to the contacted psychological teachers by express delivery, and the class was taken as the unit for collective test. Questionnaires were distributed to primary, junior and senior high schools in Guizhou, China. Common method biases are minimized through procedural control. The psychological teacher reads out the guidance in a unified way to reduce the deviation of guessing methods and control methods for the purpose of measurement (39). The teachers explained the questions that the students did not understand during the period, so as to reduce the measurement error caused by the students' understanding deviation. Before the participants filled in the questionnaires, the teachers explained the purpose and method of this study to the students, and emphasized the authenticity and confidentiality of the answers. The questionnaires were answered by the students in the classroom. At the end of the answer, the teachers collected the questionnaires and sent them back to our research team. We carefully checked the returned questionnaires and excluded those that responses, incomplete responses, unrecognized and abnormal (such as age 45).

### Data analysis

SPSS v25 software was used to primary analysis. The ratio of missing values was analyzed. Descriptive statistics were used to analyze the demographic characteristics and the scores of the C19P-SC and PHQ-9. The distribution of the data was analyzed by calculating the skewness and kurtosis levels of the data. Item statistics including mean score, standard deviation, and corrected item-total correlation.

Mplus v8.3 software was used for CFA, ESEM and measurement invariance (40). First, in terms of normality testing, the result showed that our data was normal. Hence, the Maximum Likelihood (ML) estimator was adopted. Then ESEM was performed to verify the structure of the C19P-SC. Using geomin oblique rotation, parameter estimation uses the maximum likelihood method. In addition, we also compared the CFA results with the ESEM results. To test the fit index of all models, select chi-square value (χ^2^), Comparative Fit Index (CFI), Tucker-Lewis Index (TLI), Root Mean Square Error of Approximation (RMSEA), Standardized Root Mean Square Residual (SRMR). CFI > 0.90, TLI > 0.90, SRMR close to (or less than) 0.08, RMSEA < 0.08 was an acceptable fitting model; CFI > 0.95, TLI > 0.95, SRMR < 0.08, RMSEA < 0.06 (41, 42). Next, we used ESEM to test the invariance of the C19P-SC across gender and time. Four models were established, that is, configural, metric, scalar and strict invariance, and gradually seek equivalence from loose to strict methods. The evaluation indicators for measurement invariance were as follows: ΔCFI < 0.010, ΔTLI < 0.010, and ΔRMSEA < 0.015 (43, 44). Finally, according to Revelle and Zinbarg (45) argue that McDonald's omega in fact provides a more accurate approximation of a scale's reliability. The McDonald's omega coefficients (46) was calculated in the Jamovi v2.3.16 software (47).

## Results

### Missing data

The missing rate of all variables in the current study was below 3%, and the full-information maximum likelihood method was used to settle the missing values (48).

### Descriptive statistics

Among the 1933 participants, 887 (45.9%) male and 1,033 (53.4%) female, the missing value was 13 (7%), and the mean age was 14.28 (range: 9–21, SD = 2.39). Four hundred (26.4%) from primary school, 321 (16.6%) from junior high school, and 1,090 (56.4%) from high school, the missing value was 12 (6%).

Then the descriptive statistics of the total scores of the participants in the C19P-SC and PHQ-9 were estimated. For the subscale of the C19P-SC, the mean score of the psychological was 19.15 (range: 5–30, SD = 5.28), psycho-somatic was 9.13 (range: 4–25, SD = 3.94), economic was 10.21 (range: 3–40, SD = 3.87), and the social was 14.74 (range: 3–25, SD = 4.29). The mean score of the PHQ-9 was 9.48 (range: 5–27, SD = 4.23), and 722 participants (37.35%) were diagnosed with possible severe depressive symptoms (cutoff score ≥ 10).

As shown in Table 1, the mean (and standard deviation) of items of the C19P-SC ranges from 1.63 to 4.04 (0.89 to 1.31), and the absolute value of skewness (kurtosis) of each item ranges from 0.055 to 1.583 (0.365 to 2.361). Therefore, the research data can be considered as an acceptable normal distribution (49). The correlation coefficients between each item and the other 19 items (i.e., the corrected Item-total Correlation) were between 0.291 and 0.635, which were lower than 0.80, indicating without multicollinearity (50).

**Table 1:** Descriptive statistics of the C19P-SC items.

**Item**	**Mean**	**SD**	**Skewness**	**Kurtosis**	**Corrected item-total correlation**	**Alpha if** ** item deleted**
1	2.96	1.22	−0.058	−1.001	0.587	0.889
2	4.04	1.12	−1.303	1.032	0.381	0.894
3	3.13	1.19	−0.210	−0.874	0.627	0.888
4	3.03	1.20	−0.105	−0.938	0.635	0.887
5	3.05	1.21	−0.117	−0.981	0.632	0.887
6	3.17	1.25	−0.216	−0.912	0.291	0.897
7	1.68	0.96	1.568	2.208	0.377	0.894
8	1.63	0.89	1.583	2.361	0.426	0.893
9	1.79	1.04	1.303	0.954	0.490	0.892
10	1.85	1.08	1.187	0.557	0.508	0.891
11	2.25	1.28	0.731	−0.650	0.516	0.891
12	2.58	1.26	0.268	−1.072	0.621	0.888
13	2.61	1.25	0.221	−1.081	0.620	0.888
14	2.59	1.31	0.289	−1.178	0.423	0.894
15	2.52	1.25	0.408	−0.902	0.590	0.889
16	2.96	1.24	−0.055	−1.045	0.628	0.887
17	3.57	1.18	−0.695	−0.365	0.441	0.893
18	3.42	1.21	−0.513	−0.645	0.456	0.893
19	2.30	1.18	0.667	−0.443	0.479	0.892
20	2.56	1.21	0.341	−0.829	0.614	0.888

### C19P-SC factor structure

We compare the applicability of CFA and ESEM to the C19P-SC to check the necessity of using ESEM. According to Marsh et al. (51), if the goodness-of-fit indices of CFA and ESEM were similar, CFA should be more parsimonious. On the contrary, if the ESEM was better than CFA, the provision of no cross-load is indeed an over restrictive condition.

As shown in Table 2, the fitting index of CFA was: χ^2^ = 1821.368, *df* = 164, TLI = 0.878, CFI = 0.895, SRMR = 0.067, RMSEA (90% CI) = 0.072 (0.069, 0.075), the fitting index of ESEM was: χ^2^ = 715.594, *df* = 116, TLI = 0.938, CFI = 0.962, SRMR = 0.023, RMSEA (90% CI) = 0.052 (0.048, 0.055). The fitting index of the CFA model is lower than 0.90, indicating that the model fits the data poorly, while the fitting indexes of the ESEM were up to the ideal level, which shows that the data and the model fit well. Since the CFA model can be nested in the ESEM, their comparison is meaningful (26).

**Table 2:** Comparison of fitting indexes between CFA and ESEM.

**Model**	***χ^2^***	***df***	**TLI**	**CFI**	**SRMR**	**RMSEA (90% CI)**
CFA	1821.368	164	0.878	0.895	0.067	0.072 (0.069, 0.075)
ESEM	715.594	116	0.938	0.962	0.023	0.052 (0.048, 0.055)
ESEM (male)	428.619	116	0.929	0.957	0.025	0.055 (0.050, 0.061)
ESEM (female)	430.293	116	0.939	0.963	0.025	0.051 (0.046, 0.056)
CFA-ESEM	1105.774	48	−0.06	−0.067	0.044	0.050 (0.021, 0.020)

χ^2^, chi-square goodness of fit; df, degrees of freedom; TLI, Tucker–Lewis index; CFI, Comparative Fit Index; SRMR, Standardized Root Mean Square Residual; RMSEA, Root Mean Square Error of Approximation; 90% CIs, 90% confidence intervals for RMSEA. Same below.

In the ESEM model, the four factors of the C19P-SC showed a low to moderate correlation (Table 3), indicating that the factors can be clearly distinguished. Marsh pointed out that because CFA fixed the cross-factor load at zero, the load of the corresponding factor was overestimated (27).

**Table 3:** Correlation between factors of the C19P-SC in CFA model and ESEM model.

**Factor**	**F1**	**F2**	**F3**	**F4**
F1	1	0.319^***^	0.524^***^	0.559^***^
F2	0.358^***^	1	0.405^***^	0.297^***^
F3	0.585^***^	0.485^***^	1	0.567^***^
F4	0.656^***^	0.437^***^	0.741^***^	1

Under the diagonal is EFA, and above the diagonal is ESEM.

*** P < 0.001.

The partial factor load within the factors of the ESEM model and the correlation coefficient between factors are lower than the CFA model, but the fit of the ESEM model to the data was higher than that of the CFA model. Therefore, the results of ESEM were more in line with the actual situation of data. But at the same time, it should be noted that the performance of some items in the ESEM model results was abnormal (Table 4), which is mainly reflected in that the main factor load of the item was lower than that of its sub-factor load, and these high-order factor loads mainly occur on the adjacent factors of the main factor.

**Table 4:** Factor loading of the C19P-S-C items on CFA and ESEM model.

**Items**	**CFA**	**ESEM**			
		**F1**	**F2**	**F3**	**F4**
1	0.738	0.691	0.024	0.002	0.061
2	0.516	0.534	−0.120	−0.019	0.059
3	0.821	0.831	0.021	0.013	−0.034
4	0.827	0.830	0.012	−0.012	0.006
5	0.783	0.732	0.009	0.080	−0.001
6	0.337	0.313	−0.018	−0.020	0.066
7	0.739	−0.002	0.803	−0.072	−0.018
8	0.808	−0.051	0.878	−0.049	0.007
9	0.797	0.032	0.745	0.052	−0.007
10	0.721	0.036	0.623	0.115	0.029
11	0.475	0.052	0.254	0.394	0.026
12	0.806	0.028	−0.019	0.862	−0.022
13	0.825	−0.004	−0.019	0.814	0.048
14	0.489	−0.086	0.086	0.297	0.270
15	0.593	0.026	0.205	0.169	0.413
16	0.753	0.067	0.013	0.063	0.685
17	0.584	−0.036	−0.081	−0.063	0.761
18	0.546	0.027	−0.029	−0.038	0.619
19	0.520	0.015	0.236	0.155	0.263
20	0.687	0.135	0.165	0.155	0.370

F1, Psychological; F2, Psycho-somatic; F3, Economic; F4, Social. Same below.

### Gender and longitudinal measurement invariance

#### Configural invariance

In the configural invariance test, various parameters are allowed to be estimated freely, and the fitting index obtained is shown in the configural invariance model in Tables 5, 6. All fitting indexes meet the requirements of psychometrics ($\chi_{\text{gender}}^{2}$ = 861.171, *df*_gender_ = 232, TLI_gender_ = 0.934, CFI_gender_ = 0.960, RMSEA_gender_ = 0.053; $\chi_{\text{Longitudinal}}^{2}$ = 1068.118, *df*_Longitudinal_ = 596, TLI_Longitudinal_ = 0.946, CFI_Longitudinal_ = 0.959, RMSEA_Longitudinal_ = 0.039), the configural invariance was established, and can be used as the baseline model for the metric invariance test.

**Table 5:** Multi-group ESEM comparison nested model fitting index (gender invariance, *N* = 1,933).

**Model**	***χ^2^***	***df***	**TLI**	**CFI**	**RMSEA (90% CI)**	**ΔTLI**	**ΔCFI**	**ΔRMSEA**
Configural invariance	861.171	232	0.934	0.960	0.053 (0.050, 0.057)	—	—	—
Metric invariance	959.889	296	0.945	0.958	0.048 (0.045, 0.052)	+0.011	−0.002	−0.005
Scalar invariance	1009.684	312	0.946	0.955	0.048 (0.045, 0.052)	+0.001	−0.003	0
Strict invariance	1153.221	332	0.940	0.947	0.051 (0.048, 0.054)	−0.006	−0.008	−0.003

ΔTLI, change in TLI; ΔCFI, change in CFI; ΔRMSEA, change in RMSEA. Same below.

**Table 6:** Multi-group ESEM comparison nested model fitting index (longitudinal invariance, *N* = 519).

**Model**	***χ^2^***	***df***	**TLI**	**CFI**	**RMSEA (90% CI)**	**ΔTLI**	**ΔCFI**	**ΔRMSEA**
Configural invariance	1068.118	596	0.946	0.959	0.039 (0.035, 0.043)	—	—	—
Metric invariance	1178.209	660	0.947	0.955	0.039 (0.035, 0.042)	+0.001	−0.004	0
Scalar invariance	1212.421	676	0.946	0.954	0.039 (0.036, 0.043)	−0.001	−0.001	0
Strict invariance	1434.018	696	0.928	0.936	0.045 (0.042, 0.049)	−0.018	−0.018	+0.006

#### Metric invariance

Based on the configural invariance model, the factor load invariance was set, namely, the factor load of the same index was equal in different gender and different measurement time points. The fitting results of gender metric invariance test (see Table 5 metric invariance model) showed that ΔCFI and ΔRMSEA were −0.002 and −0.005, respectively, which were < 0.01. Although the standard of ΔTLI = 0.011>0.01, the gender metric invariance model was also acceptable considering the results of the other two indicators. The fitting results of longitudinal metric invariance test (see Table 6 metric invariance model) show that ΔCFI, ΔTLI and ΔRMSEA were −0.004, +0.001 and 0, respectively, which are < 0.01. This result supports the establishment of the metric invariance across gender and time.

#### Scalar invariance

On the basis of the second step test, the intercept of observation variables was set equal in male and female group and two measurement time points. The fitting indices (see scalar invariance model in Tables 5, 6, respectively) indicate that the model fits well. The ΔCFI, Δ TLI and ΔRMSEA of the gender invariance model were −0.003, +0.001 and 0, respectively; the ΔCFI, ΔTLI and ΔRMSEA of the longitudinal invariance model were −0.001, −0.001 and 0, respectively, which were < 0.01. These results show that the gender and longitudinal invariance were established.

#### Strict invariance

On the basis of the third step test, the error variance was set equal. The fitting index were shown in the strict invariance model in Tables 5, 6, respectively. The strict invariance model of across gender was established (ΔCFI = −0.008 < 0.01, ΔTLI = −0.006 < 0.01, ΔRMSEA = −0.003 < 0.015). Established a partial longitudinal strict invariance model (ΔCFI = −0.018>0.01, ΔTLI = −0.018>0.01, ΔRMSEA = 0.006 < 0.015).

### Reliability assessment

The McDonald's omega coefficient of the C19P-SC was 0.897. The McDonald's omega coefficients were 0.842, 0.836, 0.778, and 0.760 for Factor 1 (Psychological), Factor 2 (Psycho-somatic), Factor 3 (Economic) and Factor 4 (Social), respectively.

## Discussion

Many applied studies have shown that the use of CFA to find that the model does not fit the data, and the ESEM method was more appropriate (27). Some researchers suggest that the ESEM model can better characterize the data (52), this research demonstrates this claim. In the present study, adolescents with depressive symptoms were selected as samples to test the validity and reliability of the COVID-19 Phobia Scale (C19P-S) in China. Previous studies have found that ESEM can overcome the problem of “too strict fitting standard” in traditional methods, and organically integrate the functions of EFA and CFA (31). The results of this study show that the ESEM model has more advantages than the CFA model. The specific performance was that higher fitting indicators are correlated with lower factors, and the reduction of factor correlation can improve the discriminative validity of the questionnaire. It shows that ESEM does provide a more flexible and reliable tool for analyzing scale structure.

In previous studies, the four-factor structure of the C19P-SC was obtained through exploratory factor analysis (9, 22–24). However, the fitting index of the model verified by CFA in this study was not good, which may be because there were many cross-load factor items in the C19P-SC in China. For the C19P-SC factor structure, we first used the CFA to test the four-factor model. The results showed that none of the CFA models reached acceptable levels. For the CFA model, the correlation between individual factors was too high, but this result may be due to the limitations of the confirmatory factor analysis method. Therefore, the ESEM model was used to further test the four-factor model of the C19P-SC. The study found that the ESEM model fitting results were good and the correlation between factors was significantly reduced to a low to moderate level. This method was considered to be one of the current effective methods to solve the limitations of CFA (26), by allowing the existence of cross-factor situations in the multi-factor model, the hypothetical multi-factor measurement model was more in line with the real situation. This presents the relationship between items and factors more realistically, but also faithfully presents the relationship between factors.

The ESEM model fits well, however, the cross-factor situation of some items was abnormal (i.e., item 11 and 15), that is, the main factor load of some items is lower than the sub-factor load. Most of the sub-factor were the neighboring factors of the main factor. For example, the load of item 11 “Coronavirus makes me so tense that I find myself unable to do the thing I previously had no problem doing” in its main factor “Psycho-somatic” was significantly lower than that of its sub-factor “Psychological,” while the load of item 15 “After the coronavirus pandemic, I do not feel relaxed unless I constantly check on my supplies at home” in its main factor “Economic” was significantly lower than that of its sub-factor “Social.” These cross-factor anomalies may be caused by the unclear distinction between the items due to the subtle differences between the main factors and sub-factors of the items. These results may also imply that some items have unclear measurement direction. The factor results of item 11 and item 15 were different from those of other studies (9, 22–24), in addition to the different statistical method (ESEM) used, it is also possible that the sample of this study is non-clinical depressed adolescents. The samples used in previous studies to verify the C19P-S were adults (9, 23, 24) and patients with anxiety disorders (22). Some studies have shown that psychological factors may be the main risk factors of depressive symptoms in adolescents (53, 54). For adolescents with non-clinical depressed, psychological problems are more important than physical problems. This may explain why the factor loadings of the 11 items are higher on the psychological factors than on the psycho-somatic factors. This suggests that from the beginning of primary school enrollment, we can conduct psychological screening for every child and adolescent and establish psychological files. Through early monitoring and other measures, the high-risk group of adolescent depression tendency can be found in advance. In addition, we should not only provide psychological counseling to adolescents with depressive symptoms in normal times, but also pay more attention to such groups during the period of COVID-19. The main body of the sentence in item 15 is more inclined to adults or people living alone, while most adolescents live with their parents or guardians. Adolescents may seldom experience “After the coronavirus pandemic, I do not feel relaxed unless I constantly check on my supplies at home.” It is also possible that the sentence order of item 15 (After the coronavirus pandemic, I do not feel……) and items 16 and 17 (After the coronavirus pandemic, I feel……, After the coronavirus pandemic, I actively……) is similar, which is easy to misunderstand by adolescents. In future studies of the C19P-SC, researchers can try to rewrite sentences for better research results. It is worthy of the attention of future researchers that the main factor loads of abnormal item 11 and items 15 in the confirmatory factor analysis results of this study and existing studies were both higher than 0.40 (22, 24), indicating that the relationship between these items and their main factors is still very significant. Although the item 11 and item 15 factor loads were abnormal in our study, we still chose to keep them, because the C19P-SC may be applicable to other samples in China. When conducting other sample studies in the future, Chinese researchers can decide whether to delete the item according to the actual situation of the study.

In addition, this study preliminarily verified the gender and longitudinal measurement invariance of the C19P-SC. It was found that strict invariance can be achieved in different groups (male and female) and partial strict invariance can be achieved at different time points, which indicates that the C19P-SC has invariance in Chinese adolescents with depressive symptoms (55). The results of single-group ESEM show that the four-factor structure of the C19P-SC fits well in the total, the male and the female sample. Meanwhile, based on the above results, the four-factor structure of the C19P-SC can be used as the baseline model for further research on the measurement invariance. The results of multi-group ESEM show that the four-factor structure of the C19P-SC in this study meets the model requirements of configural invariance. This indicates that the C19P-SC measured the same structure in male, female and different time points. On the basis of the configural invariance model, a metric invariance model with equal factor load was established. The establishment of metric invariance indicates that the potential characteristics and observed indicators of the 20 items of the C19P-SC have the same meaning in different genders and two time points. This study shows that the intercepts of the observed variables of the C19P-SC were equal, that is, the observed variables of different genders and time points have the same reference point (scalar invariance). The establishment of strict invariance indicates that the error variances of the C19P-SC measurement in different genders were equivalence. In previous studies, few researchers explored whether the four-factor structure model of the C19P-SC had measurement bias in longitudinal comparison. Millsap believed that configural, metric, scalar and strict invariance were all valid, which indicated that cross group comparison of the scale was meaningful (56). The above four invariances were established, indicating that the C19P-SC has gender and longitudinal measurement invariance in adolescents with depressive symptoms. The observation scores of the C19P-SC can be reasonably compared in different genders and time points.

The results of reliability analysis show that the Mcdonald's omega coefficients of the C19P-SC total scale and each subscale reached the expected acceptable level. The results of this study are consistent with the research of the C19P-S in other countries (9, 22–24), it provides effective support for the reliability of the Chinese version of the C19P-S.

In summary, the C19P-SC has good reliability and validity in adolescents with depressive symptoms, and can be used to assess the phobia of coronavirus in Chinese adolescents with depressive symptoms. But there are still some shortcomings in this study: First, the samples in this study did not pass the clinical evaluation, and the level of depressive symptoms may affect the results of the study. Secondly, this study is the first one to test the construct validity of the C19P-S by using the exploratory structural equation model. So it is impossible to analyze some fuzzy results in this study by comparing with the existing studies. Therefore, it is impossible to clearly provide an accurate explanation for the real reasons for the abnormal cross-factor phenomenon in these items. It is worthy of further discussion in the future.

## Conclusion

This study conducted a preliminary discussion on the psychometric properties of the C19P-SC used in adolescents with depressive symptoms in China. The results of the ESEM model provide support for the four-factor model. However, some items have cross-factor anomalies, suggesting that some items need to be further revised in future research. Thus, in future studies, it is necessary to test the C19P-SC structural validity and item performance when using the C19P-SC, and consider its deletion or modification based on item performance. In addition, the applicability of the C19P-SC in normal sample needs to be tested.

## Data availability statement

The original contributions presented in the study are included in the article/supplementary material, further inquiries can be directed to the corresponding author.

## Ethics statement

The studies involving human participants were reviewed and approved by Committee of the School of Psychology of Guizhou Normal University. Written informed consent to participate in this study was provided by the participants' legal guardian/next of kin.

## Author contributions

WC concepted the article and provided framework of the manuscript. QL and JS collected the data. TY analyzed the data and drafted the manuscript. WC offered suggestions and guidance for revising the data analysis of this manuscript. The final version was approved by WC. All authors contributed to the article and approved the submitted version.

## Funding

This research was funded by the Natural Science Research Funding Project of the Department of Education Guizhou Province (Grant No. Qian Ke He KY Zi [2021]299), Achievements of Guizhou Province Philosophy and Social Science Planning Project (21GZZD45), and Humanities and Social Science Research Project of Higher Education Institutions of Guizhou Provincial Department of Education (2022ZD006).

## Conflict of interest

The authors declare that the research was conducted in the absence of any commercial or financial relationships that could be construed as a potential conflict of interest.

## Publisher's note

All claims expressed in this article are solely those of the authors and do not necessarily represent those of their affiliated organizations, or those of the publisher, the editors and the reviewers. Any product that may be evaluated in this article, or claim that may be made by its manufacturer, is not guaranteed or endorsed by the publisher.
